# Performance, stability and operation voltage optimization of screen-printed aqueous supercapacitors

**DOI:** 10.1038/srep46001

**Published:** 2017-04-06

**Authors:** Suvi Lehtimäki, Anna Railanmaa, Jari Keskinen, Manu Kujala, Sampo Tuukkanen, Donald Lupo

**Affiliations:** 1 Tampere University of Technology, Department of Electronics and Communications, Engineering, Korkeakoulunkatu 3, FI-33720 Tampere, Finland; 2 Tampere University of Technology, Department of Automation Science and Engineering, Korkeakoulunkatu 3, FI-33720 Tampere, Finland

## Abstract

Harvesting micropower energy from the ambient environment requires an intermediate energy storage, for which printed aqueous supercapacitors are well suited due to their low cost and environmental friendliness. In this work, a systematic study of a large set of devices is used to investigate the effect of process variability and operating voltage on the performance and stability of screen printed aqueous supercapacitors. The current collectors and active layers are printed with graphite and activated carbon inks, respectively, and aqueous NaCl used as the electrolyte. The devices are characterized through galvanostatic discharge measurements for quantitative determination of capacitance and equivalent series resistance (ESR), as well as impedance spectroscopy for a detailed study of the factors contributing to ESR. The capacitances are 200–360 mF and the ESRs 7.9–12.7 Ω, depending on the layer thicknesses. The ESR is found to be dominated by the resistance of the graphite current collectors and is compatible with applications in low-power distributed electronics. The effects of different operating voltages on the capacitance, leakage and aging rate of the supercapacitors are tested, and 1.0 V found to be the optimal choice for using the devices in energy harvesting applications.

Supercapacitors, also called electric double layer capacitors, are used for energy storage in applications where high power or high cycle life is required^1^. They comprise two porous electrodes, current collectors, electrolyte and separator. Activated carbon (AC) is typically used for the porous electrode due to its high surface area^2^. Ideally, charge is stored electrostatically on the surface of the electrodes without the occurrence of electrochemical reactions. This is the reason for the high cycle life of supercapacitors compared to that of batteries. Certain materials such as metal oxides also exhibit pseudocapacitance, where fast and reversible Faradaic reactions can produce very high capacitances, although this also results in a reduced stability^3^. Some pseudocapacitance arises also in carbon materials due to surface functionalities^2^.

Supercapacitors are usually produced with rigid metal or polymer casing using architectures similar to batteries and electrolytic capacitors. However, printed supercapacitors have also been demonstrated^4^,^5^,^6^,^7^,^8^ and have potential advantages in numerous applications. For example, energy storage devices printed on flexible substrates, potentially flexible themselves, are needed in many applications of printed, distributed and ubiquitous electronics such as sensors. Particularly, systems using micropower energy harvesting from ambient light, vibrations, or other sources, require intermediate energy storage for continuous operation. Supercapacitors are well suited to such applications due to their high cycle life and the possibility of preparing them with safe and non-toxic materials^9^,^10^.

Screen printing is a suitable process for supercapacitor electrode fabrication, as it can produce in single passes the thick layers that are needed to produce devices with high capacitance. Screen-printed electrodes have been previously prepared from activated carbon^7^,^11^ as well as pseudocapacitive graphene composites^12^,^13^. Bar-coating^5^,^6^,^14^, blade-coating^10^,^15^,^16^ and dispenser printing^4^,^9^ have also been used for preparing thick supercapacitor electrodes. As the capacitance depends directly on the thickness of the active layer, it is important to be able to print layers reliably. Moreover, combining two electrodes with different electric double layer capacitances results in the smaller dominating the overall device capacitance^17^.

Aqueous electrolytes are not widely used in commercial supercapacitors due to their low maximum voltage of approximately 1 V. Aqueous electrolytes do, however, have some advantages over organic ones, namely their high ionic conductivity, ease of usage, environmental friendliness and safety, as well as increased capacitance due to added pseudocapacitance^18^,^19^. In applications such as distributed electronics, for which low component cost and safety of materials are important for device feasibility, aqueous electrolytes are a good choice. Previous studies with aqueous electrolytes in symmetric carbon-based supercapacitors report using voltages from 0.5 V to 1.2 V in the measurements^6^,^18^,^20^,^21^,^22^,^23^. The Béguin group has even succeeded in increasing the maximum operating voltage up to 1.9 V with aqueous sulphates^19^,^24^. The device stability at these higher voltages is dependent on the material purity, surface treatments and assembling the supercapacitors in an environment with low oxygen content.

In this work, we present the properties of supercapacitors prepared from screen printed activated carbon and graphite current collector electrodes on plastic substrate. The electrolyte is aqueous NaCl and the devices are assembled in ambient air. As a semi-automatic screen printer is used, there is variation in the layer thicknesses, resulting in a range of capacitances and equivalent series resistances (ESRs). That variation can, however, be utilized to examine the effects of layer thicknesses on the properties more closely. In many reports on printed supercapacitors, authors do not reveal how many samples have been produced, or if the reported values are averages or the best ones obtained in a large sample series. Here, we disclose the data on all samples used in the study. Additionally, we study the effect of the maximum voltage on supercapacitor properties in order to find an optimum voltage.

## Methods

The electrodes were screen printed on PET substrates (Melinex ST506 from DuPont Teijin Films) using a semi-automatic TIC SFC 300 DE screen printer from Eickmeyer. The current collectors (20 mm × 29 mm) were printed with Henkel Electrodag PF407C graphite ink, using a 60 t/in polyester screen (6 electrodes per sheet) and a round-edged Shore A 65 squeegee. Two layers were printed; each was first annealed at 120 °C for 10 minutes in a regular oven, with an additional anneal for 30 minutes in a convection oven at 120 °C to ensure that all solvent had evaporated. Sheet resistance was measured with the 4-point probe method^25^. The electrodes were cut from the substrate into 4 cm × 4 cm pieces which were weighed, then attached onto plastic films for alignment of the active layer print.

Activated carbon (AC) ink was formulated in-house with chitosan binder. First, 1.7 g chitosan (from shrimp shells, low viscosity, Sigma-Aldrich) was dissolved in 70 g of deionized (DI) water by stirring overnight with the addition of 0.7 g acetic acid. The chitosan solution was mixed with 30.9 g activated carbon powder (Kuraray YP-80F) and an additional 20 g DI water, to yield a thick paste. The ink was used to print 10 mm × 18 mm active electrodes on one end of the graphite current collectors using the same screen mesh and squeegee as were used with graphite. Due to the rapid drying of the aqueous ink, DI water was sprayed on the screen intermittently to slow down the drying. A single layer was printed for each electrode and dried in air at room temperature overnight. The electrodes were then weighed to obtain the AC mass.

Before supercapacitor assembly, Paramelt Aquaseal X2277 hot melt sealant was applied to the edges of the substrates and annealed at 80 °C for 15 minutes. The supercapacitors were then assembled by sandwiching two electrodes with a separator paper (Dreamweaver Silver AR40, 16 mm × 23 mm) and electrolyte, in most samples 1 M NaCl (Fluka TraceSelect > 99.999%, Sigma-Aldrich). The electrodes and separator were soaked with electrolyte before the assembly to ensure complete filling of the pores. An impulse heat-sealer was then used to seal the edges of the device. Additionally, 1 M KCl (Fluka TraceSelect > 99.9995%, Sigma-Aldrich) and 1 M NaNO_3_ (Fluka TraceSelect > 99.999%, Sigma-Aldrich) were tested as alternative electrolytes. In total, 82 samples were prepared. The electrodes were printed and samples assembled in three batches; the electrodes in each batch were sorted according to the AC mass and similar-mass electrodes paired up for the supercapacitor devices.

A Maccor 4300 workstation (Maccor Inc., USA) was used to characterize the supercapacitors. In the standard measurement program, the capacitor was charged and discharged to 0.9 V at 1 mA three times, charged to 0.9 V and held for 30 minutes, then discharged at 1 mA (capacitance determination), charged to 0.9 V, held for 1 hour (leakage current determination), and discharged. These steps were then repeated with currents 3 mA and 10 mA. At the end of the standard measurement program, cyclovoltammograms were measured at 100, 50, 10 and 5 mV s^–1^ (4 cycles each). The capacitance was calculated from the 1 mA discharge after the 30-minute voltage hold through C = –I/(dV/dt), between 80% and 40% of the maximum voltage, in accordance with IEC standard 62391–1:2006^26^. The equivalent series resistance (ESR) was calculated from the initial IR drop in the beginning of the discharge at 10 mA, after the 30-minute voltage hold, as the IEC standard recommends a higher measurement current for the ESR than for the capacitance for a given device class. The leakage current reported was taken from the last 1 h hold (approximately 4.5 h of the program run at this point).

Basic properties were determined from standard measurements on 30 samples with NaCl electrolyte. In addition to the standard measurement, electrochemical impedance spectra were measured with a Zennium Electrochemical Workstation (Zahner Elektrik GmbH, Germany). The frequency range used was from 10 mHz to 1 MHz, amplitude 5 mV, offset 0 V. Additionally, the samples were measured at offset 0.9 V after a 30-minute charging at that voltage.

Seven samples were assembled from pressed electrodes and NaCl electrolyte. KCl and NaNO_3_ electrolytes were used in eight samples each. The samples were characterized using the standard program.

Voltage tests were performed with 29 NaCl samples. First, four samples were measured with a voltage cycling program in which the capacitor was charged and held at the maximum voltage for 30 minutes, then discharged with 1 mA and held at 0 V for 5 minutes: this program was applied for maximum voltages of 0.8 V, 0.9 V, 1.0 V, 1.1 V and 1.2 V. Subsequently, the cycling program was repeated seven more times (40 30-minute cycles in total, 8 for each voltage).

To avoid effects of sequential cycling with voltage change, an additional cycling test was carried out with 13 capacitors using 0.8 V for 4 samples, 1.0 V for 4 samples and 1.2 V for 5 samples. For these samples the standard measurement program was performed using the corresponding maximum voltage, followed by charging and discharging the capacitor 100 times at 1 mA, and repetition of the routine program. For the final 12 samples, a voltage hold test was performed in which 4 samples for each voltage were held at the maximum voltage for 24 hours, performing the standard program before and after the voltage hold. All measurements were started directly after capacitor assembly.

## Results and Discussion

### Properties of NaCl supercapacitors

**Figure 1:**
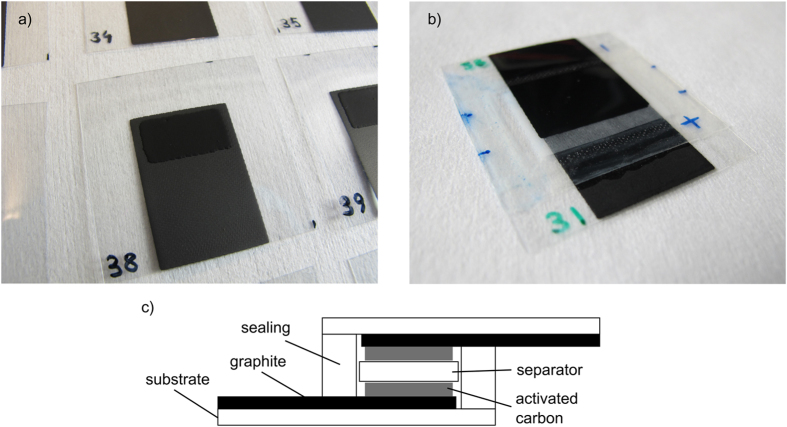
(**a**) Screen-printed electrode with graphite current collector layer and activated carbon layer. (**b**) An assembled supercapacitor. (**c**) Schematic of the supercapacitor structure.

Photographs of a printed electrode and the assembled supercapacitor are shown in Fig. 1, as well as a schematic figure of the structure. Due to the semi-manual screen printing process, the thickness of the printed layers varies from sample to sample. The graphite layers were from approximately 20 to 100 μm thick, with average sheet resistance 4.7 Ω □^–1^, ranging from 2.8 Ω □^–1^ to 6.2 Ω □^–1^ (standard deviation 0.6 Ω □^–1^). The majority of samples (75%) were in the middle of the range with sheet resistances between 4.3 Ω □^–1^ and 5.5 Ω □^–1^. The first batch of samples, used for the basic properties determination with NaCl electrolyte, had on average thicker electrodes than the subsequent printed batches due to the manual printing process. Such differences can occur, for example, due to changing the printer operator^27^.

The masses of the printed AC layers varied as well: the average mass was 6.7 mg with a range from 4.1 mg to 9.9 mg, counting the total mass of dried ink including binder. As the supercapacitors were assembled from two electrodes with similar masses, the range of total AC mass in one device ranged from 8.2 mg to 18.4 mg. SEM images of the AC layer are shown in Fig. S1 in the Supporting Information (SI). The thicknesses of the AC layers varied from 60 to 90 μm.

**Figure 2:**
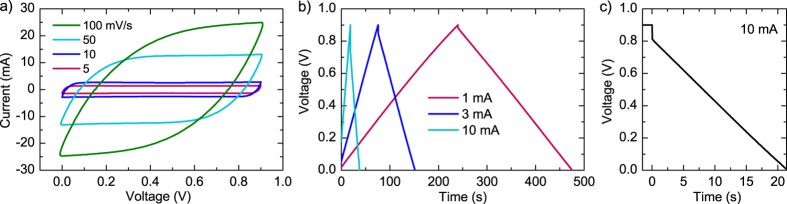
(**a**) Cyclic voltammetry, (**b**) charge-discharge and (**c**) hold-discharge (10 mA) curves of an average sample with NaCl electrolyte (C = 269 mF, ESR = 9 Ω).

Cyclic voltammetry and galvanostatic charge-discharge curves of an average sample are shown in Fig. 2a and b. The charge-discharge curves are very linear, indicative of good capacitive performance. The CV curves are for the most part fairly rectangular, but at the highest sweep rate, the resistance of the device flattens the curve.

**Figure 3:**
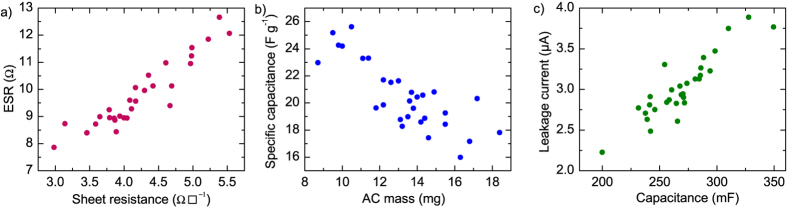
The properties of 30 supercapacitor samples with NaCl electrolyte. (**a**) ESR plotted against the average sheet resistance of the two current collectors in the device; (**b**) the specific capacitance plotted against activated carbon ink mass of both electrodes; (**c**) the leakage current measured after 1 h hold at 0.9 V plotted against device capacitance.

The equivalent series resistance (ESR) was calculated from the IR drop at the beginning of the discharge at 10 mA after holding the capacitor at 0.9 V for 30 minutes (Fig. 2c). A linear fit was made to the discharge in the linear part of voltage versus time data and the IR drop value calculated from the fitted line value at the beginning of the discharge. This eliminates a possible nonlinear voltage drop at the very beginning of the discharge, as defined in the standard^26^. The results of the 30 NaCl samples, plotted against the average sheet resistance of the two current collector electrodes, are shown in Fig. 3a.

From the graph it is clear that the ESR correlates with the sheet resistance. As the devices do not have metallic current collectors, it is reasonable to expect the contribution of the graphite electrodes to the overall ESR to be significant. The variation in current collector sheet resistances thus causes most of the variation in ESR. There was no correlation found between the ESR and the AC mass in the device, as shown in SI Fig. S2a. The values of ESR range from 7.9 to 12.7 Ω; the first batch of printed electrodes used in these devices had on average lower sheet resistances than the subsequent batches. The ESR is large compared to devices using metallic current collectors, but is still in a range which is usable in applications where the currents needed are small, e.g. < 1 mA, such as distributed sensors^10^.

The capacitance was calculated from the discharge curve measured at 1 mA after a 30-minute hold at 0.9 V. The capacitances ranged from 200 mF to 360 mF due to varying thicknesses of the AC layers; a plot displaying the capacitance as a function of total AC mass in the device is presented in SI Fig. S2b. The relatively small masses of the active layers, compared to the approximately 330-mg substrates onto which they were deposited, introduce some uncertainty in the AC mass values.

The volumetric capacitance of the supercapacitors, taking into account only the active layers, is from 9 to 10 F/cm^3^. The specific capacitance, calculated from the total AC ink dry mass (including the binder) in the device, is presented in Fig. 3b. The specific capacitance ranges from 16 F g^–1^ to 26 F g^–1^ and decreases with increasing AC mass. This implies that the entire AC layer does not effectively contribute to the capacitance for electrodes with higher mass. The densities of the active layers range from 0.4 to 0.6 g/cm^3^, and depend on the mass: electrodes with higher mass have higher density. This explains the decreasing specific capacitance with mass. Access to the pores of the activated carbon can be restricted in more tightly packed electrodes. Another possible cause of this is a poor electrical contact between the top of a thick AC layer and the current collector.

Stoller and Ruoff^28^ remark that very small active layer masses can lead to an overestimation of the specific capacitance value, and recommend test cell sizes of at least 250 mF and a mass on the order of 10 mg. This condition is fulfilled in most of the samples measured here, but the decrease of the specific capacitance with increasing mass is seen also in samples with higher mass. The maximum mass loading is limited in screen printed samples unless multiple print passes are used; moreover, very thick layers produce adhesion problems with the underlying graphite layer. The specific capacitance measured at higher currents is presented in Fig. S3a; the capacitance decreases on average 3% when increasing the measurement current from 1 mA to 10 mA.

The leakage current (float current) measured at the end of a 1-hour hold at 0.9 V ranges from 2 μA to 4 μA. To eliminate noise, a linear fit was made to the current data in the last 10 minutes of the voltage hold and its value at 1 hour taken as the leakage current. In Fig. 3c, the leakage current is shown plotted against device capacitance. The correlation with capacitance is clear as well as expected: both depend directly on the area of the carbon surface in contact with electrolyte. A common impurity causing leakage, in devices assembled in air, is oxygen which is readily adsorbed on carbon surfaces^2^ and thus its amount is directly proportional to the amount of the active material. For example, it has been shown that at the negative electrode, the mechanism of leakage is oxygen reduction to hydrogen peroxide^29^.

### Electrochemical impedance

**Figure 4:**
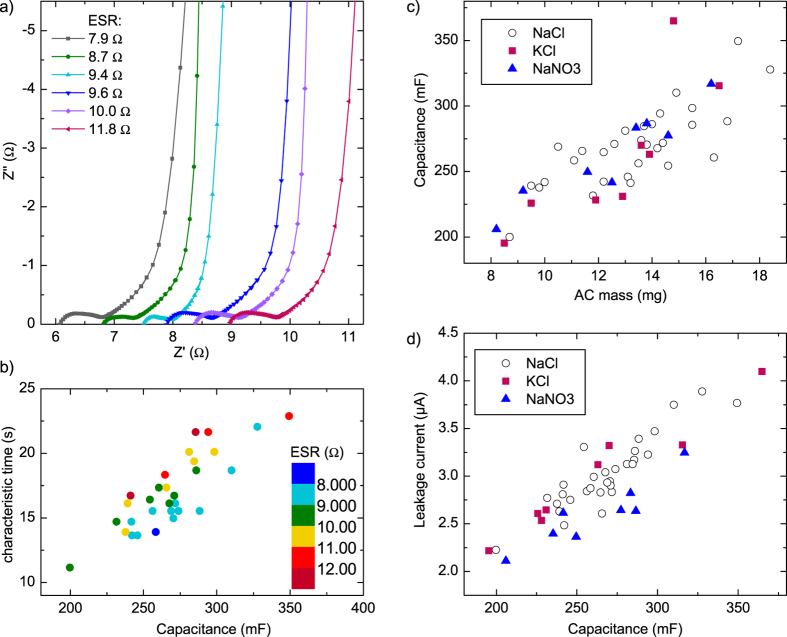
(**a**) Nyquist plots of selected samples from approximately 0.2 Hz to the high frequency range. (**b**) Characteristic time plotted against capacitance with galvanostatically measured ESR as colour scale (colour online). (**c**) Capacitance versus AC ink mass and (**d**) leakage current against capacitance for 1 M KCl and NaNO_3_ samples, compared to NaCl samples (open symbols).

Nyquist plots of selected samples are shown in Fig. 4a. At low frequencies (below approximately 1 Hz) the curves are nearly vertical, characteristic to ideal capacitance. At higher frequencies, a 45° line is observed, corresponding to capacitance and resistance distributed in a porous material [2, p. 389]. From approximately 200–400 Hz to 200–600 kHz an RC loop is found. The shape of the loop is not a perfect semicircle and the centre is below the real axis, which can be caused by distributed elements in the system as well as simultaneous processes with slightly different time constants^30^. The size of the loop stays the same when the device is measured in the charged state, at 0.9 V (not shown). From this we infer that Faradaic processes (pseudocapacitance) are not responsible for the semicircle, as the associated resistance should be voltage-dependent^2^. The loop is most likely due to an imperfect contact interface between the current collector and the active layer: Portet *et al*. have shown that an interface capacitance and resistance form a parallel RC element in series with the supercapacitive one, giving rise to a semicircle^31^.

The intercept of the left end of the semicircle with the real axis correlates well with the galvanostatically measured ESR but it is on average 2 Ω lower. Fitting a line to the low-frequency (below 0.2 Hz) part of the spectrum and extrapolating it to the real axis results in a resistance value which should correspond to the galvanostatically measured ESR^32^. This is observed here except for the highest resistances, where the value from the Nyquist plot tends to be lower (SI Fig. S4a). It is possible that when the ESR is larger, the frequency at which the device behaviour begins to resemble purely capacitive behaviour is lower, and thus the extrapolation fit should be done at even lower frequencies than those measured here.

The width of the 45° distributed resistance region was calculated from the low-frequency extrapolated value and the location of the semicircle. As it results from resistance in the active material and its pores, the distributed resistance region width should correspond to the mass or the capacitance. Some correlation is indeed observed, although there is significant variation between samples (SI Fig. S4b). Thus, it can be estimated that the components making up the total ESR of these supercapacitors are resistance of the current collectors and bulk electrolyte (leftmost intersect of the real axis, 6–9 Ω), contact resistance between the current collector and active material (semicircle width, 0.5–1.1 Ω) and distributed resistance in the porous active material (45° segment, 0.8–1.5 Ω). As was observed in the ESR dependence on sheet resistance, most of the device ESR is caused by the current collectors. Consequently, the best way to improve the devices would be to improve the conductivity of the graphite layer.

The complex capacitance can be calculated from the impedance data at each frequency through C′ = –Z″/(2πf|Z|^2^) and C″ = Z′/(2πf|Z|^2^), where C′ and C″ are the real and imaginary parts of the complex capacitance^33^. Example plots of the complex capacitance data are shown in SI Fig. S5a. The maximum of the real part of capacitance, C′, is found at the lowest frequency and should correspond to the capacitance measured galvanostatically^33^. This is also found here (shown in SI Fig. S5b).

The imaginary part, C″, is related to energy dissipation in irreversible processes^33^, and has a maximum at some frequency f_0_. The reciprocal of this frequency is a characteristic time, τ_0_, of the system, and it has been described as a relaxation time^33^, or a measure of how quickly the capacitor can be discharged with over 50% energy efficiency^34^. As the data points are sparse towards the low-frequency end, a spline fit was made to the C″ data to obtain a better estimate of the maximum of C″ (SI Fig. S6).

The characteristic time found for these samples ranges from 11 s to 23 s, increasing with capacitance (Fig. 4b). The correlation of τ_0_ with capacitance is easily understood as both are directly related to the amount of charge in the capacitor. Using the ESR of the samples as a colour scale in the plot, it can be seen that τ_0_ depends not only on capacitance, but also increases with increasing ESR. As τ_0_ is related to the losses in the device, and thus the power, it can be expected that the ESR contributes to it as well. To reduce the energy loss when the resistance increases, a slower discharge with a smaller current is needed. The galvanostatic discharge time at 10 mA for an average sample (C = 266 mF, ESR = 10.1 Ω) was 21.0 s, well above its characteristic time of 17.3 s.

### Effect of the electrolyte

Two different electrolytes were investigated as substitutes for NaCl: KCl and NaNO_3_, with 8 samples of each type. The results plotted against AC mass, are presented in Fig. 4c. It can be seen that there is no clear difference between the electrolytes in terms of capacitance, compared to variation between individual samples. In previous studies, it has been found that KCl yields a higher capacitance than NaCl in aqueous supercapacitors^35^,^36^. In this work, the large variation between samples, as well as the large ESR of the devices, mask small differences in device performance.

The leakage current, which was found to increase linearly with capacitance in NaCl samples, also does so with the other two electrolytes (Fig. 4d). The differences between electrolytes are not substantial, but NaNO_3_ yields a somewhat lower leakage than the other two for a given capacitance value. It is thus possible that the Cl^−^-ion contributes to the reactions causing leakage current. Based on this small test, changing the electrolyte to NaNO_3_ instead of NaCl could be justified, although further testing with a larger number of samples as well as other electrolyte options would be beneficial. As the differences observed here were small, NaCl was used in the subsequent voltage test.

### Voltage test

The maximum voltage of a supercapacitor is an important parameter which determines what applications the device can be used in. Moreover, as the energy stored in a capacitor is proportional to the square of the voltage, it is important to be able to use the highest possible voltage. The main limitation to the voltage is the electrochemical window of the electrolyte, which for water is approximately 1.2 V. Another factor is an increase in the pseudocapacitance contribution with increasing voltage, which has been reported for carbon supercapacitors^37^.

In the first experiment, we charged four supercapacitor samples with NaCl electrolyte to five different voltages from 0.8 V to 1.2 V, holding the device at each upper voltage for 30 minutes prior to discharge at 1 mA. After the 1.2 V hold and discharge, the cycles were repeated again seven more times starting from 0.8 V (see SI Fig. S7a for the whole test procedure). The capacitance was calculated from each discharge curve in accordance with the IEC standard^26^.

**Figure 5:**
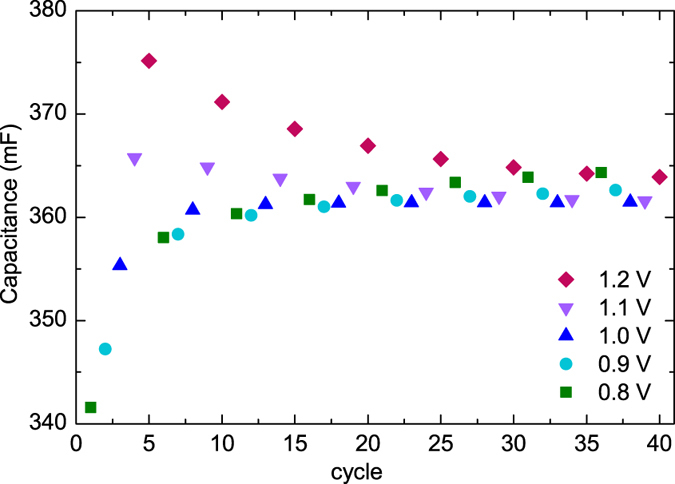
Capacitance calculated from discharge after 30 min hold at upper voltage: upper voltages cycled eight times from 0.8 V to 1.2 V at 0.1 V intervals. Total duration of measurement 33 h.

The capacitance plotted against cycle number for one sample is shown in Fig. 5. It can be seen that the apparent initial difference between capacitance for different maximum voltages (in the first five cycles) is quite drastic. However, the capacitor was held in the charged state for 30 minutes in each cycle, so capacitor aging plays a significant role here as well. When the cycling is continued, the capacitance values measured at 0.8 V and 0.9 V keep increasing, but the ones at 1.1 V and 1.2 V decrease. Towards the end of the approximately 33-hour measurement, the capacitances measured at different voltages converge. The results from the other three samples are similar and differ only in the speed of this convergence. It is difficult to separate effects of the cycling from the voltage change; for example, whether the low-voltage capacitance improvement is due to cycling at higher voltages in between. Ruiz *et al*.^37^ report a similar finding of increased capacitance with voltage but do not mention if the previous measurement rounds of the same sample at different voltages affect the results.

To distinguish the effect of the cycling from the effect of changing the voltage, three sample sets were measured at voltages 0.8 V, 1.0 V and 1.2 V. The samples were cycled 100 times galvanostatically, with the routine measurement program performed before and after the cycling (SI Fig. S7b). Another test was done for another set of similar samples but instead of cycling, a 24-hour voltage hold at the maximum voltage was used (SI Fig. S7c). It has been shown that a voltage-hold test results in quicker aging than cycling, and is thus more reliable for predicting supercapacitor stability^38^.

**Figure 6:**
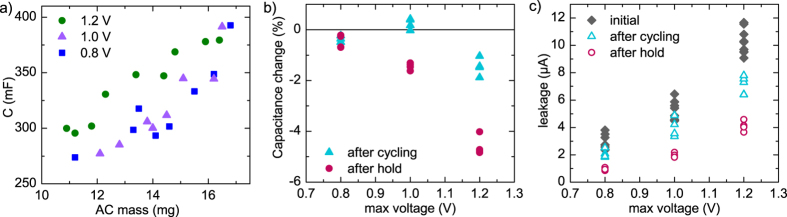
(**a**) Initially measured capacitances of samples measured at different voltages, plotted against AC ink mass. Each data point is a different sample. (**b**) Change in capacitance after cycling and after voltage hold at different voltages; separate sample batches were used for the cycling and the hold. (**c**) Leakage current at different voltages initially (all samples), after cycling and after voltage hold (separate samples).

The initial capacitance results at the different voltages are shown in Fig. 6a and the specific capacitance in SI Fig. S8. As with previous samples, the range of AC layer thicknesses results in a range of capacitances, but it is clear that for a given AC mass, the capacitance is considerably higher at 1.2 V. There is not a significant difference between the capacitances at 0.8 V and 1.0 V. The most probable reason for the increase at 1.2 V is pseudocapacitance, which is voltage-dependent. Because the AC ink was prepared in ambient air, as were the capacitors, a significant contributor to the pseudocapacitance is oxygen. It has been reported that pseudocapacitance is the reason for increasing capacitance with increasing voltage and it can account for up to 40% of measured device capacitance^37^. In this case, most pseudocapacitive reactions occur at a device voltage higher than 1.0 V.

The relative change in capacitance for each sample is shown in Fig. 6b. At 1.2 V, the capacitance decreases on average 1.5% during the cycling test and 4.6% during the hold test, consistent with the findings of Weingarth *et al*.^38^ that a hold test causes faster aging, especially when holding the supercapacitor above the rated voltage. It should be noted that in the cycling test there is also a voltage hold of approximately 4.5 h in total during the routine measurement segment, which contributes to capacitor aging. For 1.0 V, the change in capacitance was on average + 0.2% in the cycling test and –1.4% in the voltage hold test. For some reason, the capacitance appears to very slightly improve in the cycling test at this voltage. At 0.8 V, the capacitance decreases on average 0.4% in the cycling test and 0.5% in the voltage hold test. It may be that at 1.0 V, some initial reactions occur which increase the capacitance, for example through better surface wetting (because of changes to surface groups) or some other process, while 0.8 V is too low a voltage for such reactions to occur. At 1.2 V, the degradation is so much faster that this behaviour cannot be seen. The slight improvement at 1.0 V is probably related to the initial improvement of capacitance at lower voltages observed in the voltage cycling test (Fig. 5).

The leakage current of the cycling and voltage hold test samples, compared to initial values, are shown in Fig. 6c plotted against the maximum voltage and in SI Fig. S9a plotted against capacitance. The relative change in leakage is plotted against the maximum voltage in SI Fig. S9b. The difference between the voltages is significant: the initial leakage current is three times higher for samples measured at 1.2 V compared to those measured at 0.8 V. The dependence on voltage can be expected for leakage that originates from Faradaic reactions^39^. The leakage current falls during capacitor aging, which can be explained by Faradaic reactions of impurities that are depleted as the aging continues. The fall is approximately 30% in the cycling test for all voltages and approximately 60% in the voltage hold test. This is consistent with the faster aging observed in voltage hold versus cycling tests^38^.

### Performance potential in applications

The applications envisioned for the supercapacitors presented here are those where low cost, non-toxicity, cyclability and flat shape are desired. Distributed and ubiquitous electronics can be used to sense and control various parameters from wearable electronics measuring body signals to devices monitoring conditions in buildings; energy harvesting from the ambient environment can be used to power such devices autonomously, but it requires a supercapacitor or a battery as an energy buffer. Upscaling the printing process with a fully automated system enables more consistent layer thicknesses to be produced^40^.

The supercapacitors can be modified depending on the requirements for the stored energy content and output power. For example, if a 100 mV voltage loss was acceptable, the ESR of 10 Ω would allow discharge current of 10 mA. With 1 V potential, the maximum usable power^1^ is then 25 mW. Without changes in the choice of materials, the output power could be further increased by optimizing the geometry through shortening and widening the current collectors or by applying a thicker current collector ink layer.

Since the environments of use for printed supercapacitors are diverse, the requirements for packaging and other structures can vary strongly for different supercapacitors, making comparisons difficult. However, our electrical properties compare well with those reported by other groups for supercapacitors on flexible substrates. Lechêne *et al*.^41^ have fabricated printable supercapacitors on stainless steel current collectors with specific capacitance of about 100 mF/cm^2^ and ESR of about 40 Ωcm^2^, compared to our values of 150 mF/cm^2^ and 18 Ωcm^2^. For supercapacitors made using conductive carbon fabrics, Jost *et al*.^7^ report specific capacitances of 180 and 440 mF/cm^2^ with ESR values in of 50 and 14 Ωcm^2^. In both cases, gel electrolytes with harmful chemicals are used, which necessitates a further encapsulation of the device. The supercapacitors presented here can be used as prepared in any environment, because the materials are non-toxic and thus present no danger even in the case of a mechanical breakage. The aqueous electrolyte makes the use of a metallic current collector in the long term challenging due to corrosion issues, but the ESR value reached with carbon-only materials is feasible for applications with low current demands.

The thickness of the supercapacitors was approximately 600–700 μm in the center with the AC electrode layers, and thinner at the edges, down to 125 μm at the corners with only one layer of substrate. The average device volumetric capacitance is then 0.39 F/cm^3^. The relatively thick PET film was chosen for its good qualities as printing substrate and its good heat resistance; however, the barrier qualities of the film are not very good. We have previously reported results on the optimization of the substrate so that longer-term operation is also possible^8^.

The energy storage capacity of a 0.3 F supercapacitor depends on the used maximum potential according to the formula E = ½ CV^2^. If the supercapacitor is discharged to 0.4 V, maximum voltages of 1.2 and 0.8 V correspond to 192 mJ and 72 mJ released energy, respectively. Despite the higher energy available with higher maximum voltage, it is beneficial to use lower voltage if, for example, the energy harvesting system has very low power output, since in that case the leakage current at the higher voltage may consume a large percentage of the harvested energy. If the supercapacitors are connected in series to obtain higher voltage without balancing, from the reliability point of view it is advisable not to exceed 1 V for one cell in a series connection since slight differences in the capacitance and leakage current can result to unbalanced voltage distribution over the supercapacitors.

## Conclusions

Symmetric supercapacitors fabricated with screen-printed AC electrodes and aqueous NaCl electrolyte were characterized. The semi-automatic printing process yielded a range of layer thicknesses which resulted in ESR values from 7.9 Ω to 12.7 Ω and capacitances from 200 mF to 360 mF. Leakage currents measured after 1 h hold at 0.9 V were from 2 μA to 4 μA. The specific capacitance of the devices was on average 20 F g^–1^, calculated from total device capacitance and total AC ink dry mass. The specific capacitance was found to decrease with increasing AC mass. Possible poor contact in thicker AC layers was examined by pressing the printed layer, but no difference was found to unpressed electrodes.

Impedance spectroscopy was used to analyse the components of the resistance. As there was no metallic current collector, the resistance of the printed graphite current collector was the main component of ESR, contributing 6–9 Ω, with contact resistance between graphite and AC as well as the distributed resistance inside the pores causing the rest of the ESR.

Testing the supercapacitors at different voltages revealed that the capacitance increases when increasing the voltage from 1.0 V to 1.2 V: the reason is added pseudocapacitance which becomes significant at that voltage. On the other hand, the leakage current and degradation rate also increase significantly with voltage. The rated voltage of a supercapacitor should be as high as possible to maximize the energy, but a compromise of 1.0 V is the best choice when using the supercapacitors in an application such as an energy harvester.

## Additional Information

**How to cite this article**: Lehtimäki, S. *et al*. Performance, stability and operation voltage optimization of screen-printed aqueous supercapacitors. *Sci. Rep.* **7**, 46001; doi: 10.1038/srep46001 (2017).

**Publisher's note:** Springer Nature remains neutral with regard to jurisdictional claims in published maps and institutional affiliations.

## Supplementary Material

Supplementary Information
