# Supplementary material for: Performance, stability and operation voltage optimization of screen-printed aqueous supercapacitors
Source: Sci Rep. 2017 Apr 6;7:46001. doi: 10.1038/srep46001 (PMC5382703; doi:10.1038/srep46001)
Supplement: Supplementary Information [file srep46001-s1.pdf]

Supporting Information

Performance, stability and operation voltage optimization of screen-printed aqueous supercapacitors

Suvi Lehtimäki[a,*], Anna Railanmaa[a], Jari Keskinen[a], Manu Kujala[a], Sampo Tuukkanen[b], Donald Lupo[a]

[a] Tampere University of Technology, Department of Electronics and Communications Engineering, Korkeakoulunkatu 3, FI-33720 Tampere, Finland
[b] Tampere University of Technology, Department of Automation Science and Engineering, Korkeakoulunkatu 3, FI-33720 Tampere, Finland

[*] Corresponding author

suvi.lehtimaki@tut.fi, donald.lupo@tut.fi

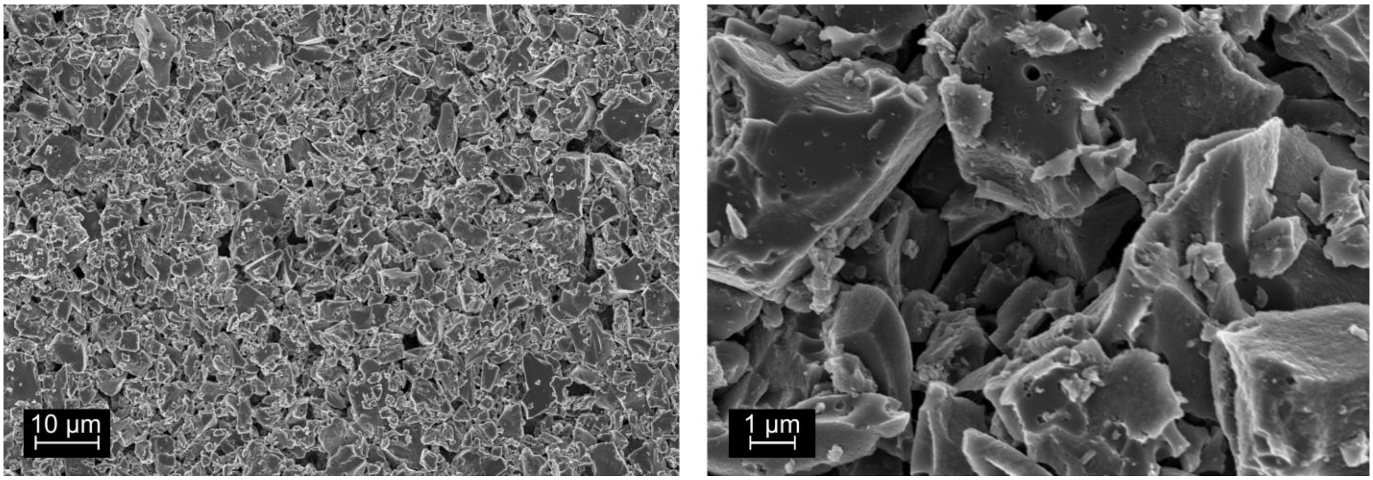

Fig. S1. SEM images of the activated carbon layer at 2 000 x and 20 000 x magnifications (3 kV).

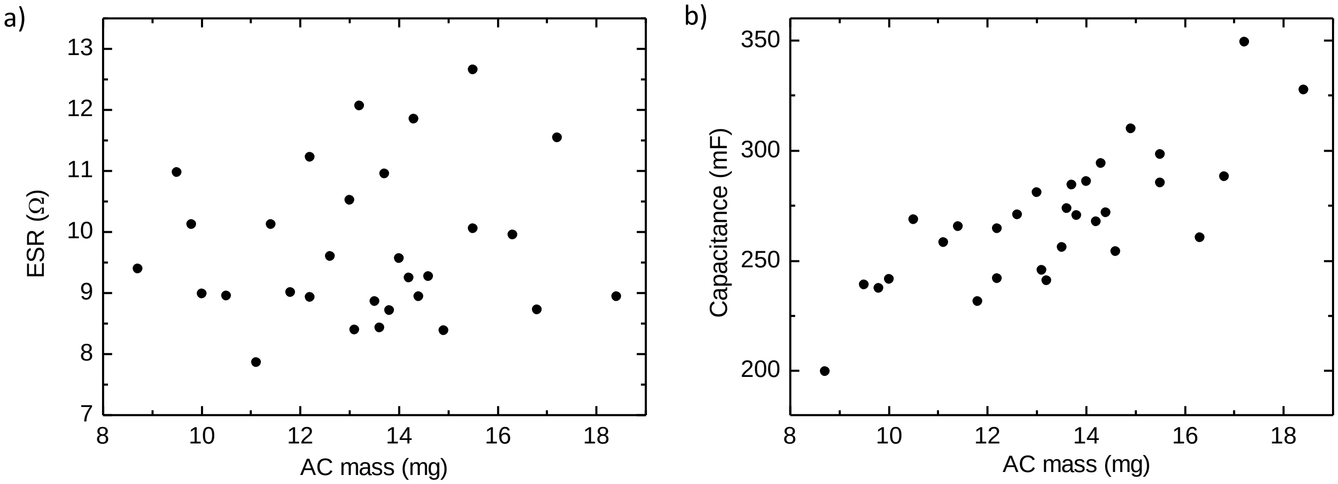

Fig. S2. a) The ESR and b) the capacitance of 30 supercapacitor samples plotted against total AC mass in the device.

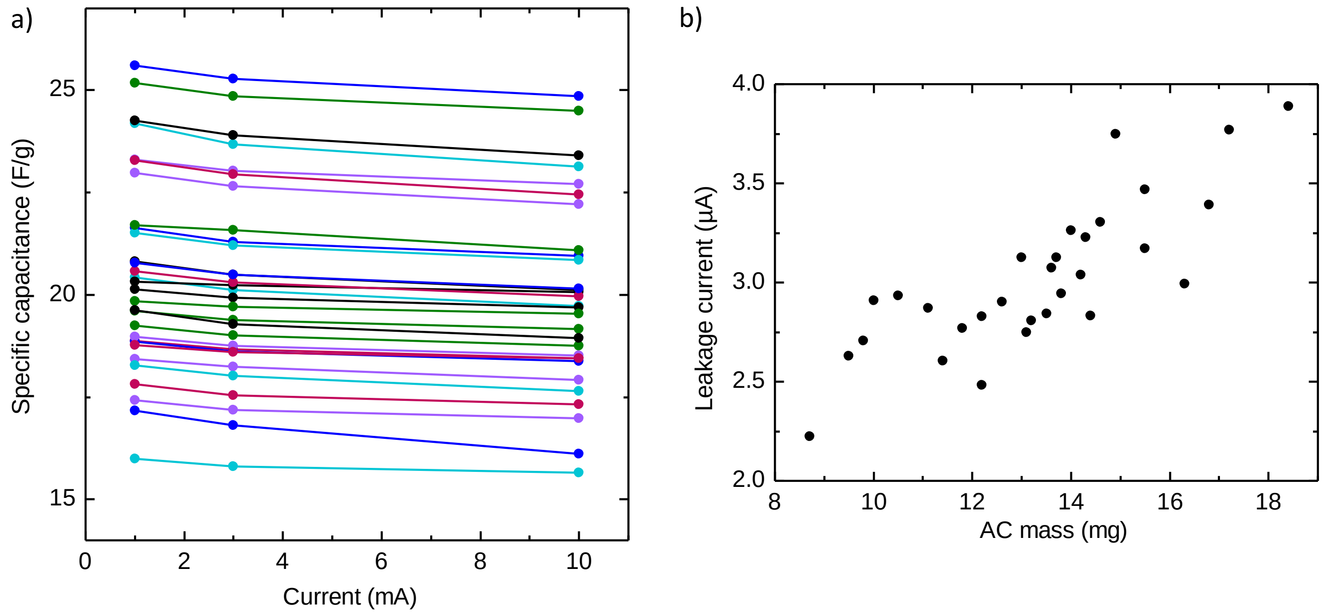

Fig. S3. a) The specific capacitance of the supercapacitors measured at 1, 3 and 10 mA discharge currents after a 30-minute voltage hold. b) The leakage current of 30 supercapacitor samples plotted against the total AC mass in the device

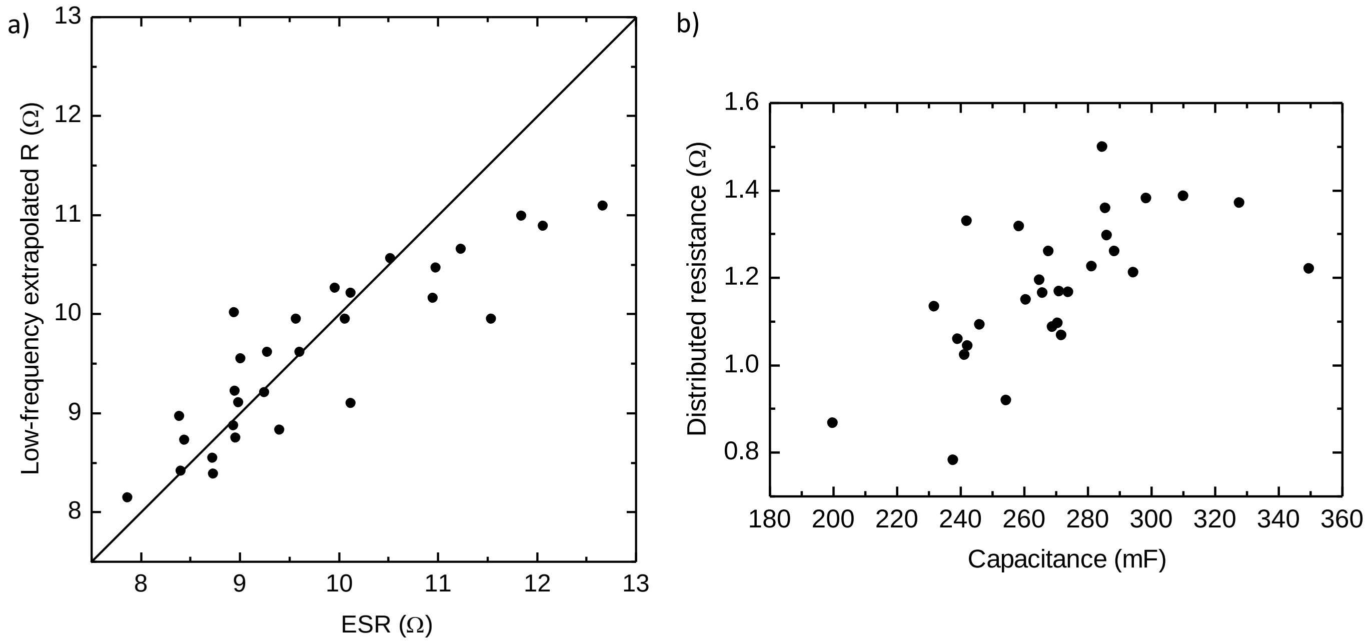

Fig S4. a) Intersection of the extrapolated Nyquist plot at low frequencies with the real axis, plotted against galvanostatically measured ESR. Line indicates equal resistance values. b) The width of the distributed resistance region in Nyquist plots plotted against capacitance.

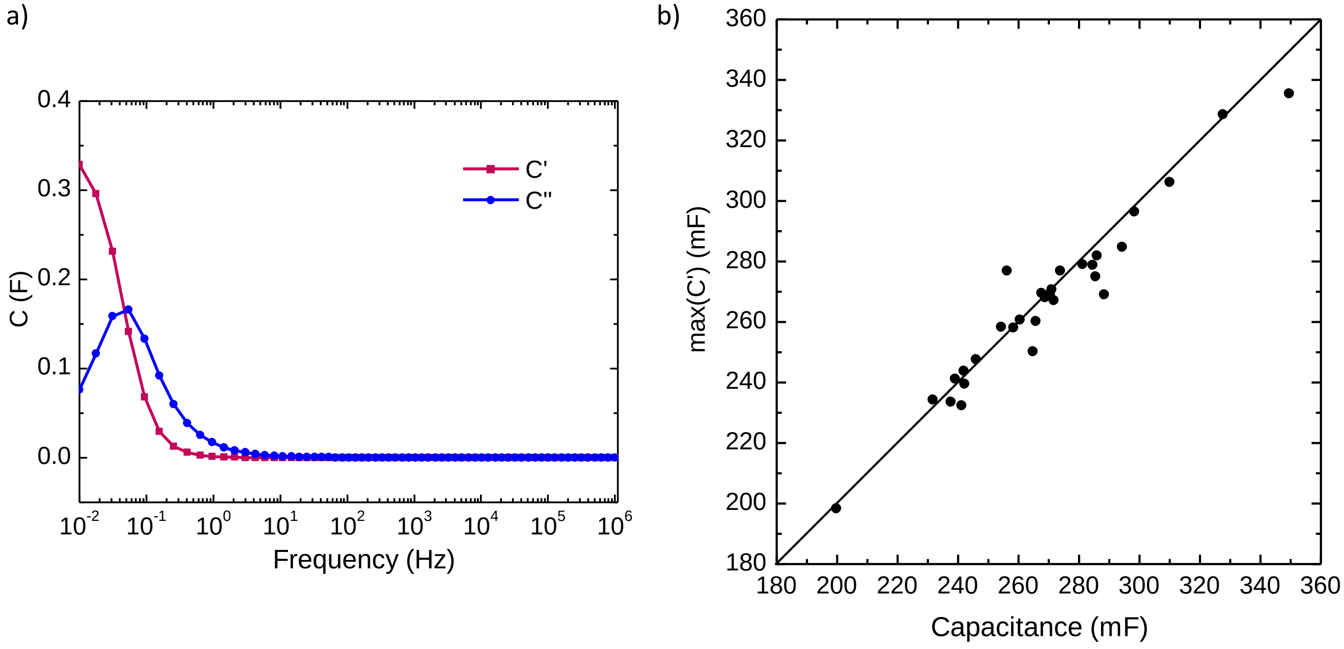

Fig. S5. a) Complex capacitance real (C') and imaginary (C'') components for one sample (C=328 mF), plotted against frequency. b) The maximum of C' plotted against galvanostatically measured capacitance. Line indicates equal capacitance values.

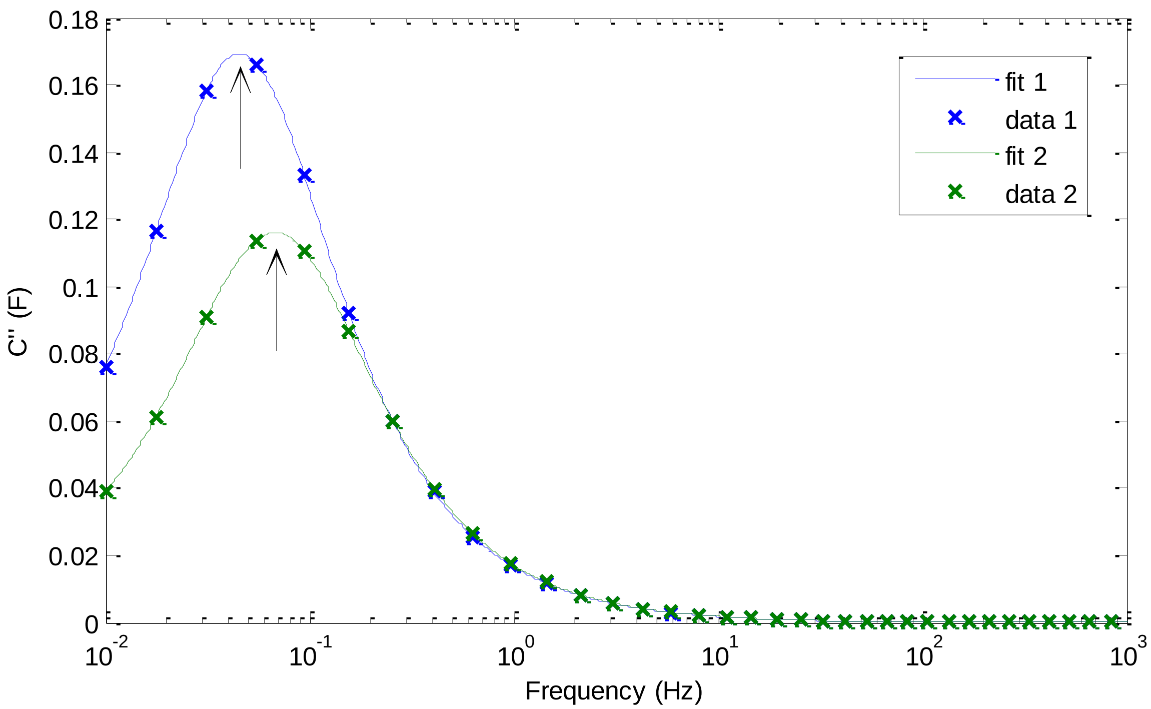

Fig. S6. Two examples of spline fits to C'' data. Without the fit, the maxima from the two data sets would appear to be at the same frequency.

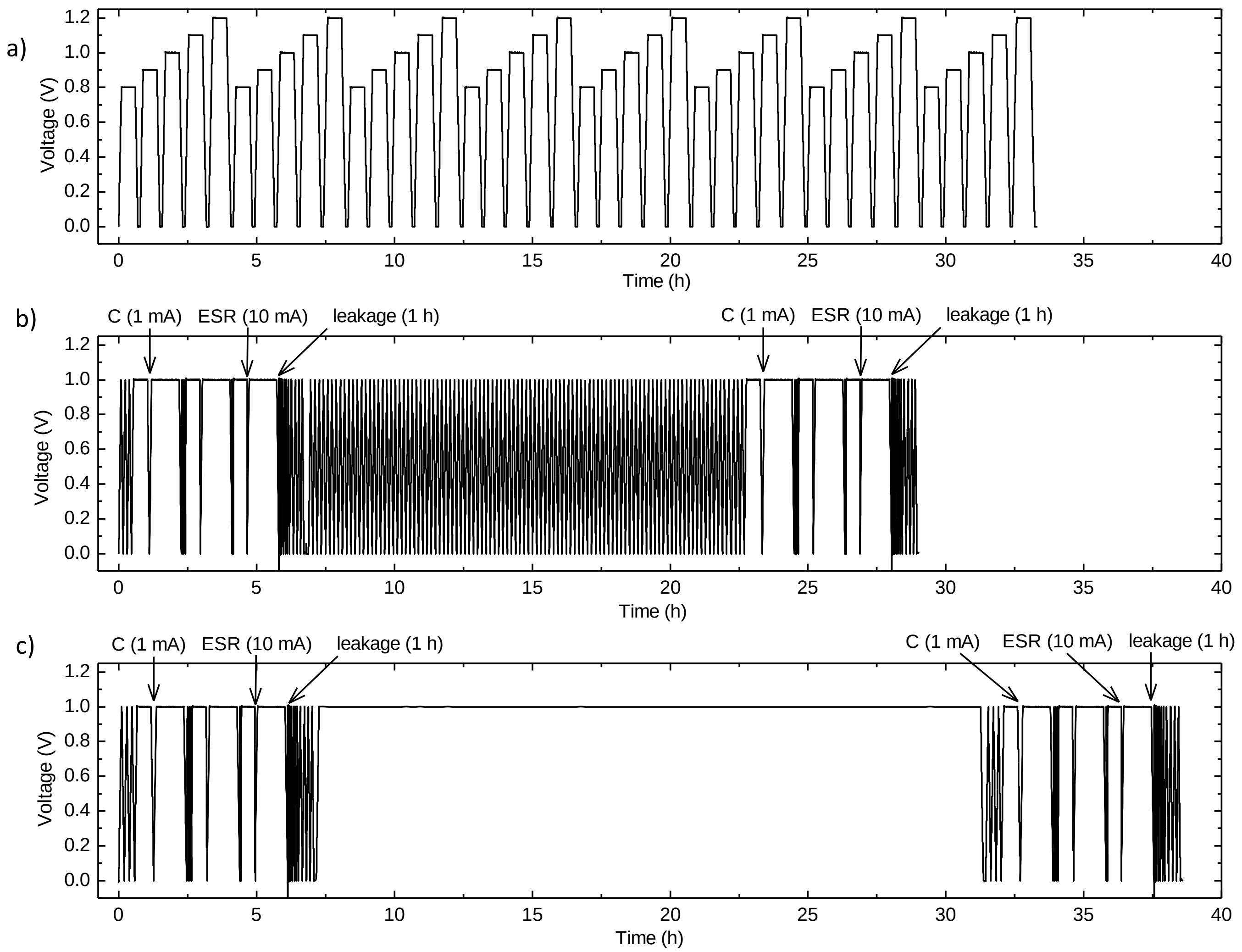

Fig. S7. The test procedures for a) voltage-cycling test and the b) cycling test and c) voltage hold test with the routine measurement program at the start and end.

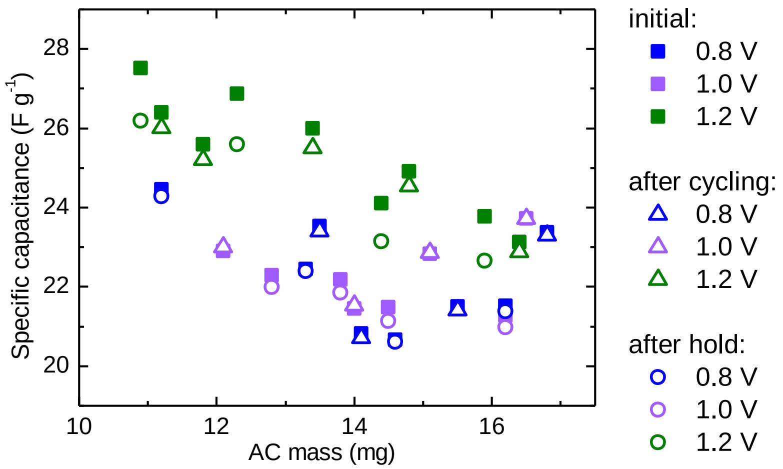

Fig. S8. Specific capacitance plotted against AC mass for the different voltages. Different sample sets were used in the cycling and hold tests.

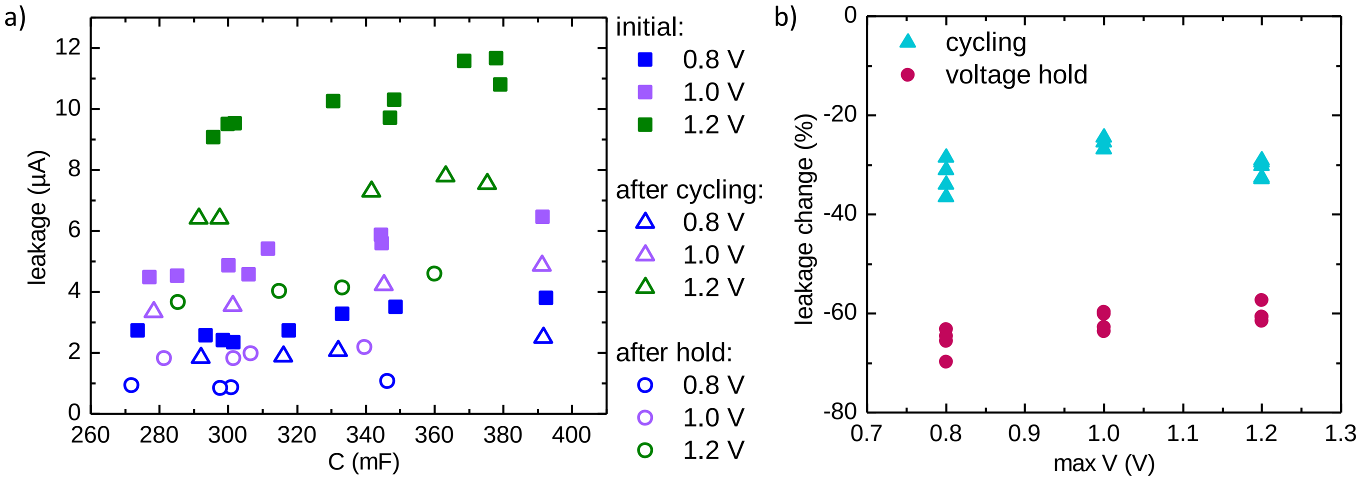

Fig. S9. a) Leakage current plotted against capacitance for the different voltages. b) Change in leakage current as percent from the initial value at different voltages. Different sample sets were used in the cycling and hold tests.
